# Exploring the impact of GLP-1 analogues and DPP-4 inhibitors on cognitive decline in elderly patients with type 2 diabetes: a competing risks analysis from a real-world cohort

**DOI:** 10.3389/fphar.2026.1822158

**Published:** 2026-10-15

**Authors:** Vera Battini, Giacomo Biganzoli, Federica Di Pierro, Annamaria Pia Terlizzi, Francesca Vittoria Rizzi, Domenica Ancona, Patrizia Boracchi, Carla Carnovale

**Affiliations:** 1 Pharmacovigilance and Clinical Research, International Centre for Pesticides and Health Risk Prevention, Department of BioPharmacovigilance & Clinical Research, Intmedical and Clinical Sciences (DIBIC), ASST Fatebenefratelli-Sacco University Hospital, Università degli Studi di Milano, Milan, Italy; 2 Medical Statistics Unit, Department of Biomedical and Clinical Sciences L. Sacco, “Luigi Sacco” University Hospital, Università degli Studi di Milano, Milan, Italy; 3 Pharmaceutical Department, ASL BT, Trani, Italy

**Keywords:** cognitive impaiment, cumulative risk, dipeptidy peptidase-4, elderly population, glucagon like peptide 1 (GLP-1)

## Abstract

**Background:**

Older adults with type 2 diabetes (T2DM) face an increased risk of cognitive decline. While glucagon-like peptide-1 receptor agonists (GLP-1RAs) have shown neuroprotective effects, comparative evidence versus dipeptidyl peptidase-4 inhibitors (DPP-4i) in real-world older populations is limited.

**Methods:**

We conducted a retrospective cohort study using health administrative data, including individuals aged ≥65 initiating GLP-1RAs or DPP-4i therapy between 2010 and 2018. Patients with prior cognitive disorders were excluded. The primary outcome was a composite of incident cognitive disorders or death. Analyses were adjusted for clinical and demographic characteristics. Marginal structural models and competing risks methods were used to estimate treatment effects. Both an ‘As treated’ (AT) and an ‘Intention to Treat’ (ITT) designs were employed.

**Results:**

A total of 2,579 individuals were included (GLP-1RAs: 393; DPP-4i: 2,186), with a median follow-up of 44 months in the AT analysis. GLP-1RAs users had a lower risk of the composite outcome, especially in the early follow-up (HR at 2 years: 0.5 [95% CI: 0.26–0.93]). Competing-risk analysis showed a lower early risk of death without prior cognitive disorders among GLP-1RA initiators. The point estimate of sub-distribution HR was 0.4 also for cognitive disorders, yet not statistically significant because of the small number of events.

**Conclusion:**

GLP-1RAs use was associated with a lower early risk of the composite outcome of cognitive disorders or death compared with DPP-4i in older adults with T2DM. The observed association with cognitive disorders alone was directionally consistent with a potentially lower risk, but the very small number of events precludes firm conclusions regarding a protective effect on cognitive outcomes. Longer follow-up and cognitive-specific evaluations are needed to confirm these associations.

## 1 Introduction

Robust evidence indicates a complex interplay between diabetes-related factors, such as insulin resistance and chronic inflammation, and cognitive decline. Cognitive disorders often manifest as deficiencies in attention, processing speed, memory, and executive function. Furthermore, the presence of vascular complications exacerbates cognitive decline in diabetic patients (Aderinto et al., 2023). Studies have revealed that the prevalence of major cognitive disorders in type-2-diabetes mellitus (T2DM) patients ranges from approximately 15%–25%, while the prevalence of mild cognitive impairment ranges from approximately 30%–60% (Srikanth et al., 2020). Given this interplay between central nervous system functions and glucose homeostasis, antidiabetic drugs are getting increased attention for a potential drug repurposing for the treatment of neurodegenerative diseases (Battini et al., 2023a; Battini et al., 2023b).

GLP-1 receptor agonists (GLP-1RAs) may exert neurobiological effects through central and peripheral pathways, though their ability to cross the blood–brain barrier (BBB) remains debated. Preclinical data suggest variable BBB penetrance, via circumventricular organs, diffusion, or receptor-mediated transport, while human relevance is uncertain. Cognitive and psychiatric effects may also stem from peripheral mechanisms, such as immune, metabolic, or gut–brain axis modulation, with BBB disruption in neuropsychiatric conditions adding further complexity (De Giorgi et al., 2025a).

Clinically, this hypothesis remains speculative, as cognitive benefits of GLP-1RAs are still unclear in trials involving psychiatric and neurological patients. However, an increasing number of observational studies suggest potential cognitive benefits in diabetic patients, supported by preclinical and mechanistic evidence of neuroprotective and anti-inflammatory effects (De Giorgi et al., 2025a; De Giorgi et al., 2025b).

Dipeptidyl peptidase-4 inhibitors (DPP-4i), a pharmacological class closely related to GLP-1RAs, remain comparatively less explored. DPP-4i prolong the half-life of endogenous GLP-1 by inhibiting the DPP-4 enzyme, though this indirect mechanism is considered a weaker mechanism than direct receptor activation. Overall, the interaction between DPP-4i and cognitive function remains complex and incompletely understood. Linagliptin, which does not cross the BBB and is a P-glycoprotein substrate, likely exerts neuroprotective effects peripherally (Mousa and Ayoub, 2019; Bernstein et al., 2023). However, pathological alterations in BBB integrity, such as those observed in Alzheimer’s disease, may enhance central penetration (Srinivas, , 2015). In contrast, the lipophilic omarigliptin appears able to cross the BBB (Ayoub et al., 2018). These findings suggest superior neuroprotection with GLP-1RAs compared to DPP-4i, but evidence remains limited.

In older adults with T2DM, mortality as a competing risk (which occurs when death from any cause prevents a person from experiencing another specific event of interest, *i.e.* a non-fatal medical outcome) may underestimate cognitive burden and treatment effects. To address this gap, we aimed to compare the impact of DPP-4i and GLP-1RAs on a composite outcome of death or cognitive disorders, whichever occurred first, providing a broader and clinically relevant assessment in a frail, real-world population. Subsequently, we explored the incidence of cognitive disorders and death without prior cognitive disorders separately, to better understand the drugs influence on aging and cognitive health. These findings may help guide therapeutic choices in elderly patients, where balancing metabolic control and neurological outcomes is a key clinical challenge.

## 2 Methods

### 2.1 Study population, study design and data source

The data used for the present study were retrieved from the healthcare utilization databases of Puglia, a region in southern of Italy that accounts 4 million people. Each Italian citizen, at birth or immigration, is assigned a personal civil registration number (Codice Fiscale), which serves as a unique identifier in national administrative databases. This number enables access to comprehensive, virtually lifelong information on demographic characteristics, cause of death, claimed prescriptions at community pharmacies and hospital discharges, according to diagnoses and procedures coded as in the International Classifcation of Diseases, Ninth Revision, Clinical Modification (ICD-9-CM) system. All Italian citizens have equal access to healthcare services as part of the National Health Service. To preserve privacy, in the analyses of the regional databases each individual identification code is automatically de-identified, the inverse process being allowed only to the Regional Health Authority upon request from judicial authorities.

The primary analysis was designed to estimate the effect of continuous treatment exposure; therefore, it followed an ‘As-treated’ design. Under this framework, patients were classified according to their initial treatment and were followed until subsequent treatment discontinuation or switching. To test the robustness of our findings against potential informative censoring due to differential discontinuation rates, the effect of treatment initiation was also explored using an Intention-to-Treat (ITT) analogue design. In this framework, patients were classified based on their initial treatment assignment and were followed until the end of the study period, regardless of subsequent treatment discontinuation or switching. For our study, we retrieved from the healthcare utilization databases all citizens living in the province of Barletta-Andria-Trani (Italy) and who were 65 years and older when redeeming the first prescription for GLP-1RAs [Anatomical Therapeutic Chemical (ATC) system: A10BJ] or DPP-4i [ATC code: A10BH] between 1st January 2010 and 31st December 2018. At present, the official Italian therapeutic indication for these two drug classes is only T2DM. The study population was tracked through the administrative registries from the date of the first redeemed prescription for the drugs of interest, known as the index date (Figure 1). The study population was then divided into two cohorts: individuals treated with DPP-4i and those treated with GLP-1RAs.

**FIGURE 1:**
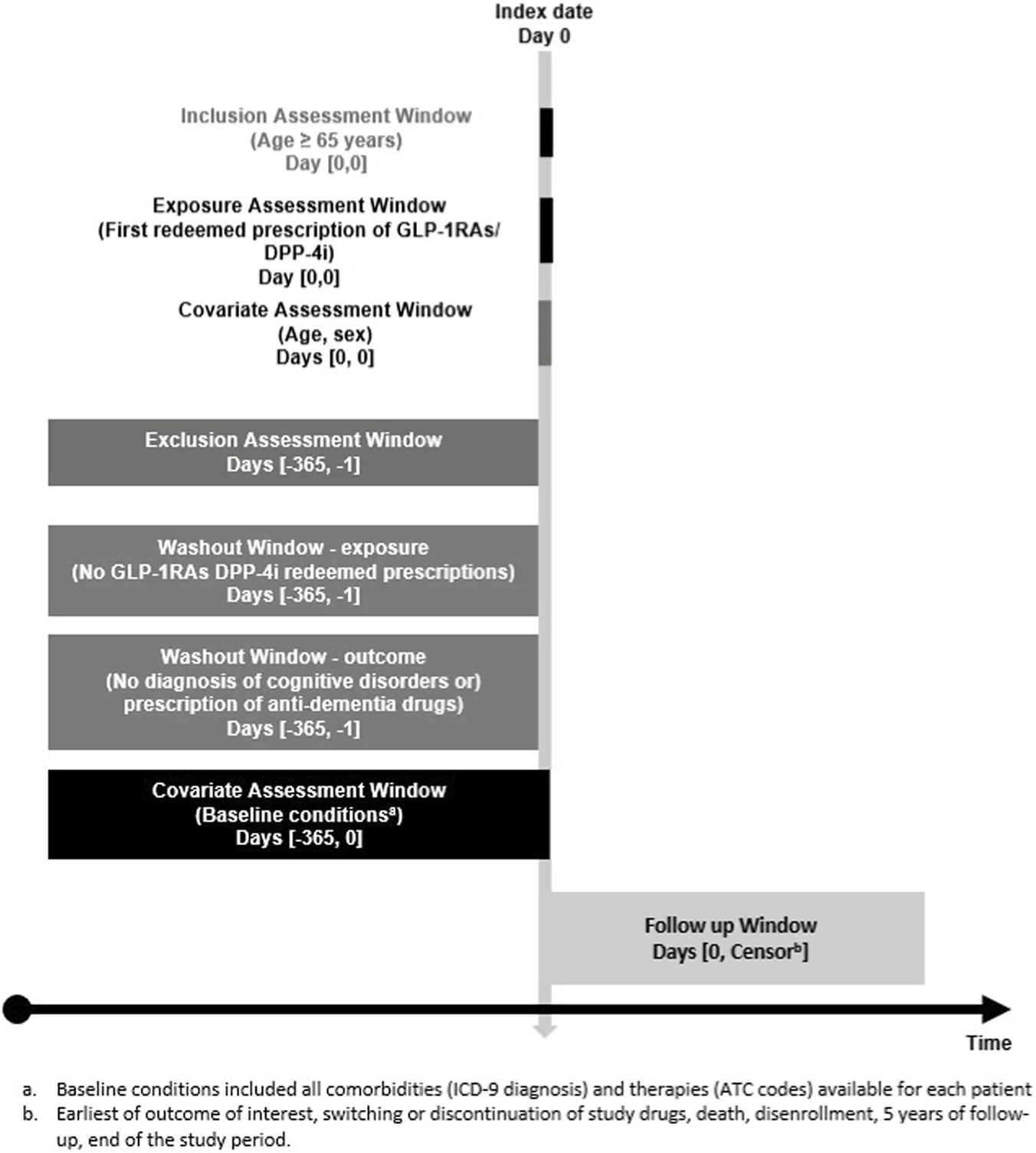
Study design and cohort selection. The study population consisted of individuals aged ≥65 years with type 2 diabetes mellitus who initiated treatment with glucagon-like peptide-1 receptor agonists (GLP-1RAs) or dipeptidyl peptidase-4 inhibitors (DPP-4i) between 1 January 2010 and 31 December 2018. Individuals with evidence of cognitive disorders before treatment initiation, missing relevant covariate information, or not meeting the eligibility criteria were excluded. The index date was defined as the date of the first redeemed prescription for GLP-1RAs or DPP-4i. Abbreviations: DPP-4i, dipeptidyl peptidase-4 inhibitors; GLP-1RAs, glucagon-like peptide-1 receptor agonists.

### 2.2 Follow-up

Individuals in the two cohorts were followed for Five years from the index date until the occurrence of the outcome or were censored in case of emigration, the end of follow-up, death, or the end of data coverage. In the ‘As-treated’ framework, censoring also applied to individuals who switched from one treatment to the other or discontinued it.

The period 2010–2018 for registered prescriptions was chosen to ensure a minimum of 5 years of follow-up for all included subjects. The decision for a 5-year follow-up period was based on previous studies (Battini et al., 2024; Tang et al., 2025; Tang et al., 2024; Rea et al., 2023).

### 2.3 Outcomes

The outcome of main clinical relevance was hospital discharges with a primary or secondary diagnosis of cognitive disorders. The list of the ICD codes used are available in Supplementary Table S1. The validity of diagnoses of major cognitive disorders in the Italian registers has been previously assessed, with a positive predictive value of 59% (Affinito et al., 2024).

### 2.4 Statistical analysis

The primary goal of our analysis was to evaluate the effect of DPP-4i vs GLP-1RAs on a composite endpoint, defined as either time to death or early onset of cognitive disorders, whichever occurred first. This helped us assess the causal effect of drug exposure on the probability of experiencing a first failure event, which is a clinically meaningful measure in a population facing multiple health threats.

We then examined the two components of the composite outcome separately: the onset of cognitive disorders, and death without any evidence of cognitive disorders. Given the advanced age of the cohort, we expected that mortality would significantly affect the likelihood of observing cognitive disorders onset. Moreover, we anticipated a correlation between the timing of cognitive disorders onset and death. Simply treating death as a censoring event, as is common in classical proportional hazards models, could therefore introduce bias in estimating the cause-specific incidence of cognitive disorders, especially when inverse probability of censoring weighting (IPCW) techniques cannot ensure that time to censoring for the competing event is conditionally independent of time to cognitive disorders, given covariates (Satagopan et al., 2004; Coemans et al., 2022; Howe et al., 2011).

To address this issue, we aimed to estimate the causal effect of drug exposure on the cause-specific cumulative incidence function (CIF) for cognitive disorders, along with its associated hazard, *i.e.* the sub-distribution hazard (SDH). The CIF represents the probability over time that a specific event, such as cognitive disorders or death without cognitive disorders, occurs as the first event. The SDH function models the instantaneous rate of occurrence of that specific event, accounting for the presence of competing events. By regressing the SDH on covariates, we can make direct and robust inferences about the CIF (Coemans et al., 2022).

Given the discrete nature of our follow-up data, we used a grouped version of the Fine and Gray proportional sub-distribution hazards model (Berger et al., 2020). This approach, which falls within the generalized linear model (GLM) framework, uses a binomial estimation scheme weighted for the sub-distribution hazard. Covariates, such as GLP-1RAs versus DPP-4i therapy, were linked to the discrete hazard using a log or complementary log-log (clog-log) link function. This modeling strategy provided several advantages. We modeled the baseline SDH (for the reference level of the covariates) using regression splines, which enabled us to compute smoothed CIFs conditional on covariates. From these CIFs, we derived clinically relevant measures of treatment effect, including relative risk (RR), absolute risk difference (ARD), and relative risk difference (RRD) at 2 and 4 years of follow-up, improving interpretability (Sutradhar and Austin, 2018).

When graphical inspection indicated a violation of the proportionality assumption, we relaxed this by including interaction terms between covariates and the baseline hazard function. This allowed us to model time-varying treatment effects rather than assuming constant hazard ratios over time. Since the study was observational in nature, the treatment groups were not directly comparable in terms of causal interpretation. To address this, we used marginal structural models (MSMs), incorporating individual weights into GLM. These weights adjusted for imbalances in clinical and demographic characteristics between the treatment groups. Individual weights were constructed using the following logistic model:

$$logit\left[P\left(Drug=GLP-1Vage,sex,X\right)\right]=\beta_{0}+\beta_{1}age+\beta_{2}sex+X\beta^{\tau }$$

where “X” is a matrix of dummy variables indicating the presence and the frequency of ICD-9 and medication codes selected following a high-dimensional Propensity Score (hdPS) approach.

Namely, we followed the procedure described in Karim et al. (Karim, 2025), identifying two main sources of proxy data linked to the primary dataset: ICD-9 codes and medication data recorded in the year prior to therapy initiation. We excluded known surrogates of exposure or outcome and filtered codes based on their prevalence, retaining ICD-9 codes and medications appearing in at least 10 and 15 patients, respectively. For each retained code, we generated three recurrence indicators:

- Occurred at least once,
- Occurred more frequently than the median,
- Occurred more frequently than the 75th percentile.

Using the Bross formula, we ranked these variables based on their estimated biasing potential (log absolute bias) and selected the top 20.

For the clinical characteristics standardized mean difference (SMD) was computed to assess whether was as close as possible to 0 and well below 0.1.

In additional sensitivity analyses, reported in the Supplementary Material, we included the calendar year of treatment initiation in the weighting model to account for underlying secular trends in prescribing practices.

### 2.5 Governance and transparency

Access to databases was provided by the Pharmaceutical Department of ASL BT, Trani, Italy. Compliance with national and European laws on personal data was guaranteed by the Pharmaceutical Department through the generation of unique anonymous codes for each patient.

Due to its observational design and use of anonymized data, this study was exempt from ethics review. There was no direct patient or public involvement in study design, analysis, or reporting. Participants under 16 years were excluded. Informed consent could not be obtained, as the retrospective study used routinely collected data for policy and public health purposes.

## 3 Results

Within the period 2010-2018 we obtained ICD-9 data of 4,761 individuals who started a lowering glucose therapy with GLP-1RAs or with DPP-4i. 2,141 individuals were excluded from the analysis since they were less than 65 years old. 36 individuals were removed since they were hospitalized for a cognitive disorders-related events (23) or had a drug-prescription for cognitive disorders in the year before the start of the therapy (11). Nine individuals have missing values in age covariate thus were excluded from the analysis.

In the end, we filtered 2,186 (85%) individuals with a DPP-4i prescription and 393 (15%) individuals with a GLP-1RAs prescription. During the follow-up, 92 individuals, 31 from GLP-1RAs and 61 from DPP-4i, changed the therapy. At the end of the follow-up, 1942 did not experienced any event, 90 had onset of cognitive disorders, while 554 died without evidence of neurodegenerative disease. The main ICD-9 code retrieved and considered as the outcome of interest was 290.40 (vascular dementia, uncomplicated), with 30 events in the DPP-4i group and 1 event within the GLP-1RA group. During the 5-year follow-up period, 29 subjects were prescribed pharmacological treatments for dementia in the DPP-4i group compared with only two patients in the GLP-1RA group. In the AT analysis, median follow-up time was 44 months (3.6 years), with a median (95% C.I.) follow-up of 55 (49–60) months among DPP-4i users and 18 (14–23) months among GLP-1RA users. The shorter follow-up among GLP-1RA users mainly reflects the differential discontinuation of the medication rates. In the ITT analysis, whose results are reported in the Supplementary Material, median follow-up time was well above 60 months for both the groups.

Treatment groups were different with respect to age, proportion of gender, concomitant medications such as Metformin, SGLT2 inhibitors, Insulin. Table 1 reports the mean value of the variables within groups as well as the standardized mean difference between groups, before and after IPW.

**TABLE 1:** Mean values of the demographic, comorbidities and concomitant medications within GLP-1RA and DPP-4i and standardized mean difference between GLP-1RA and DPP-4i. Values before and after IPW are reported.

Category	Characteristic	Before IPW			After IPW		
		GLP-1RA	DPP-4i	SMD	GLP-1RA	DPP-4i	SMD
Demographics	Age, years	70.0	75.3	0.978	74.3	74.6	0.055
Demographics	Male sex	45.7%	48.7%	0.061	50.8%	48.3%	0.048
Comorbidity burden	Charlson index (score)	0.0	0.1	0.148	0.1	0.1	0.063
Healthcare use	Distinct drug classes (polypharmacy)	0.7	0.6	0.208	0.7	0.6	0.081
Healthcare use	Hospitalizations, prior year	0.1	0.1	0.135	0.1	0.1	0.118
Comorbidities	Myocardial infarction	0%	0.6%	0.114	0%	0.6%	0.112
Comorbidities	Congestive heart failure	0%	0.6%	0.114	0%	0.6%	0.109
Comorbidities	Cerebrovascular disease	0%	0.6%	0.114	0%	0.6%	0.108
Comorbidities	Mild liver disease	0%	0.2%	0.068	0%	0.2%	0.068
Comorbidities	Peptic ulcer disease	0%	0.1%	0.053	0%	0.2%	0.056
Comorbidities	Peripheral vascular disease	0.3%	0.1%	0.031	0.2%	0.1%	0.026
Comorbidities	Malignancy (non-metastatic)	0.3%	0.5%	0.030	0.2%	0.4%	0.047
Concomitant medications	Metformin	31.8%	18.4%	0.312	24.9%	20.2%	0.113
Concomitant medications	SGLT2 inhibitors	2.8%	0.6%	0.166	1.2%	0.8%	0.041
Concomitant medications	Insulin	25.9%	20.8%	0.121	23.2%	21.6%	0.039
Concomitant medications	Thiazolidinediones	1.7%	2.6%	0.066	3%	2.5%	0.033
Concomitant medications	Antiplatelet/anticoagulant	4.7%	6%	0.057	7.4%	5.7%	0.066
Concomitant medications	Antipsychotics	1.1%	0.9%	0.024	0.5%	0.9%	0.044

DPP-4i, dipeptidyl peptidase-4, inhibitors; GLP-1RAs, glucagon-like peptide-1, receptor agonists; IPW: Inverse probability weighting; SGLT-2: Sodium-glucose cotransporter 2; SMD: Standardized mean difference.

### 3.1 Time-to-first failure (composite event)

During the follow-up period, 634 individuals experienced the composite outcome, defined as either the onset of cognitive disorders or death without prior evidence of cognitive disorders. Among these, 593 were in the DPP-4i group and 41 in the GLP-1RAs group.

Figure 2 shows the cumulative risk of experiencing the composite event, estimated with the Kaplan-Meier method. The risk curve for the GLP-1RAs group remains consistently lower than that of the DPP-4i group throughout the follow-up, indicating a lower overall probability of experiencing either event in those treated with GLP-1RAs.

**FIGURE 2:**
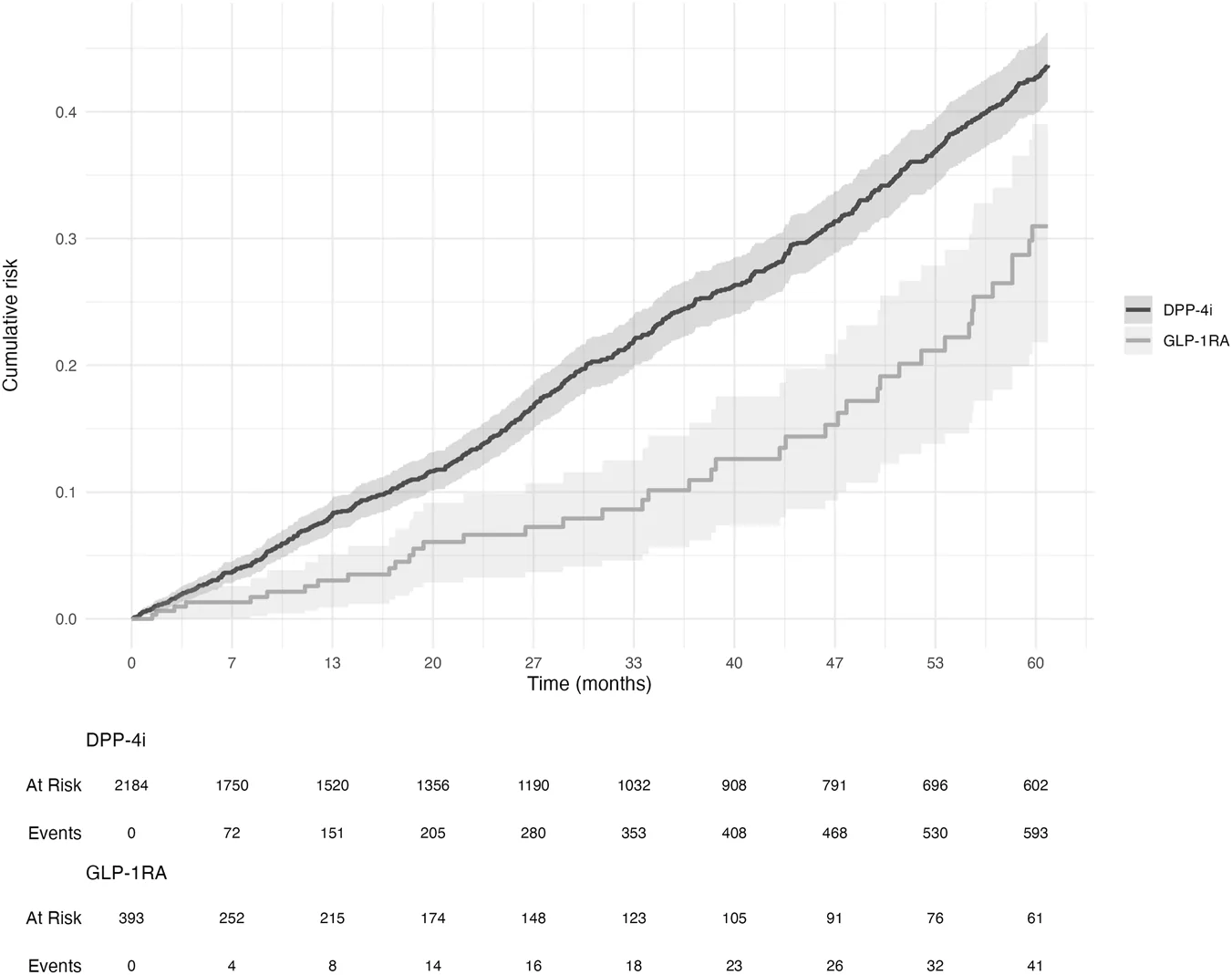
Unadjusted Time-to-first failure analysis. A first failure was defined as the occurrence of either a cognitive disorder or death without a prior diagnosis of cognitive disorder, whichever occurred first. In unadjusted estimates of cumulative risks of a first failure for DPP-4i and GLP-1RAs groups, computed with the Kaplan-Meier estimator. Abbreviations: DPP-4i, dipeptidyl peptidase-4 inhibitors; GLP-1RAs, glucagon-like peptide-1 receptor agonists.

Figure 3B shows the cumulative risk curves predicted from the marginal structural model, along with clinically relevant measures of treatment effect at 24 and 48 months. At 24 months, the risk for individuals treated with GLP-1RAs is 34% that of those treated with DPP-4i, corresponding to an absolute risk reduction of 9% and a relative risk reduction of 66%. At 48 months, the risk is 72% that of the DPP-4i group, with a 9% absolute risk reduction and a 28% relative risk reduction.

**FIGURE 3:**
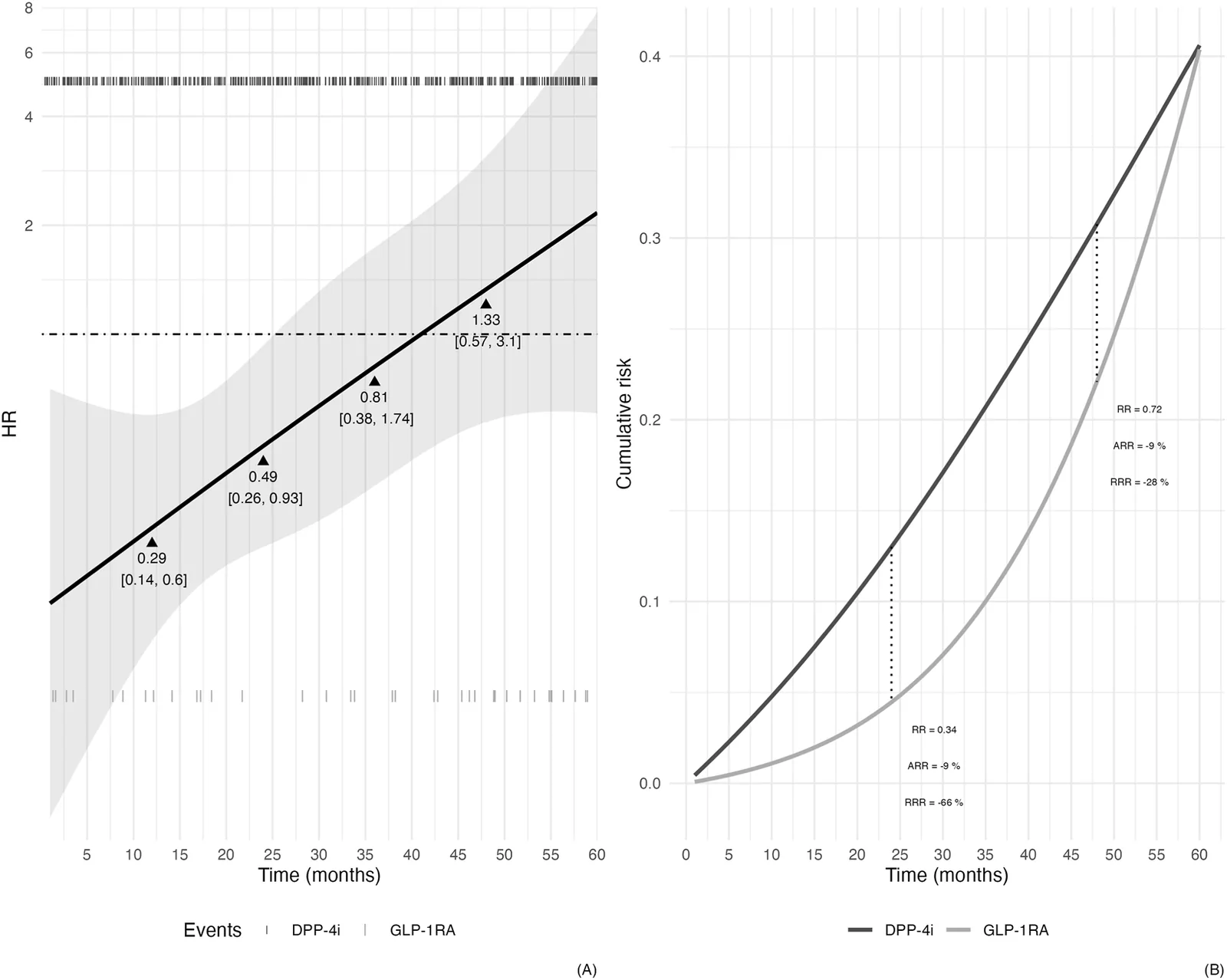
Adjusted Time-to-first failure analysis. A first failure was defined as the occurrence of either a cognitive disorder or death without a prior diagnosis of cognitive disorder, whichever occurred first. In **(A)**, time-varying hazard ratio estimated with a marginal structural model. In **(B)**, adjusted estimates of cumulative risks of a first failure for DPP-4i and GLP-1RAs groups and the related effect measures computed at 2 and 4 years of the follow-up. Abbreviations: DPP-4i, dipeptidyl peptidase-4 inhibitors; GLP-1RAs, glucagon-like peptide-1 receptor agonists; HR: hazard ratio.

Figure 3A presents the time-varying hazard ratio for GLP-1RAs versus DPP-4i, modelled using restricted cubic splines with three knots. The time-varying hazard ratio gradually increases from values well below zero -HR [95% C.I.], 0.3 [0.14, 0.6], 0.5 [0.26, 0.93], and 0.81 [0.38, 1.74] at 1, 2 and 3 years of follow-up respectively-, eventually crossing the value of 1 toward the end of the 5-year follow-up - HR [95% C.I.], 1.33 [0.57, 3.1]-. The shaded area represents the 95% confidence intervals. Assuming no residual confounding, these results suggest that GLP-1RAs therapy is associated with a significant protective effect against the composite outcome in the earlier phases of follow-up. However, this protective effect decreases over time and is no longer evident by year five.

Overall, these findings indicate that GLP-1RAs therapy may offer a time-dependent protective effect against cognitive disorders or death, with the most pronounced benefits observed within the first few years of follow-up.

The ITT results (Supplementary Figures S1, S2) are in line with these main results. The early protection of GLP1-RA for the composite endpoint is still maintained statistically significant in the first Two years of the follow-up when this design is employed. Furthermore, the sensitivity analyses including calendar year of treatment initiation (Supplementary Figures S7, S8; Supplementary Figures S13, S14) in the weighting model were also in line with the main results presented here.

### 3.2 Competing risks

In the Five years of follow-up, the overall risk of developing cognitive disorders as a first event was low—reaching a maximum of 6%—especially when compared to the risk of death, which rose to 37%.

Figure 4 shows the marginal cumulative incidence curves for cognitive disorders onset in both the DPP-4i and GLP-1RAs groups. Individuals treated with GLP-1RAs consistently exhibited a lower risk of cognitive disorders as a first event. However, the estimates are imprecise due to the small number of events observed in this group—only 4 cognitive disorders cases in the GLP-1RAs cohort compared to 85 in the DPP-4i group. Because of this limited number of events, we were unable to model a time-varying effect of GLP-1RAs on the sub-distribution hazard for cognitive disorders onset. Instead, we applied a standard grouped proportional sub-distribution hazard model (Figure 5A). Despite the small event count, the results suggest a strong association: the sub-distribution hazard ratio (SDHR) was 0.4, even though, the difference was not statistically significant, as the confidence interval for the SDHR included 1 and extended up to 1.2.

**FIGURE 4:**
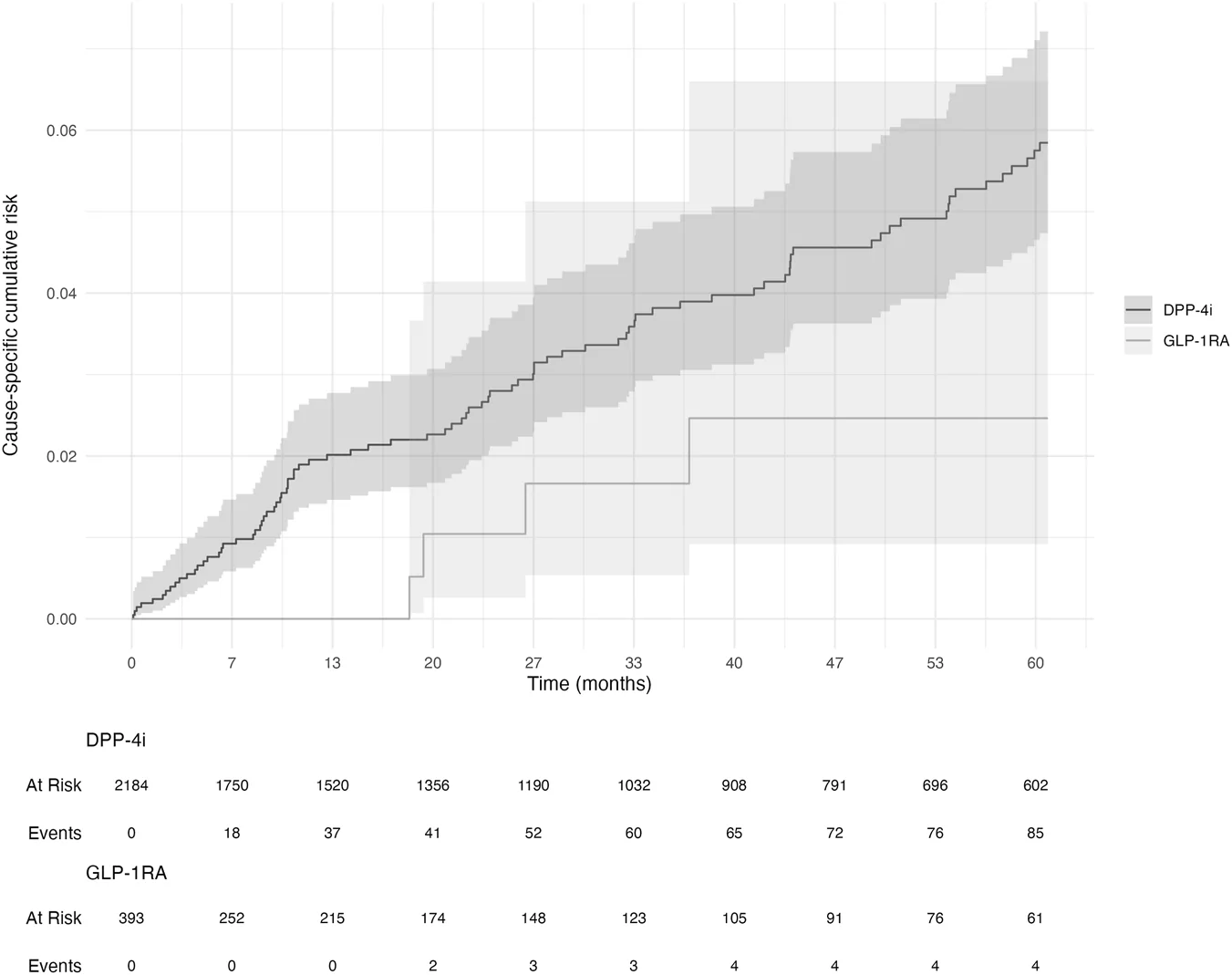
Unadjusted competing-risk analysis of cognitive disorders. Cognitive disorder was considered the event of interest, while death without a prior cognitive disorder was treated as a competing event. In unadjusted estimates of crude cumulative estimates of cognitive disorder as a first event for DPP-4i and GLP-1RAs groups, computed with the Aalen Johansen estimator. Abbreviations: DPP-4i, dipeptidyl peptidase-4 inhibitors; GLP-1RAs, glucagon-like peptide-1 receptor agonists.

**FIGURE 5:**
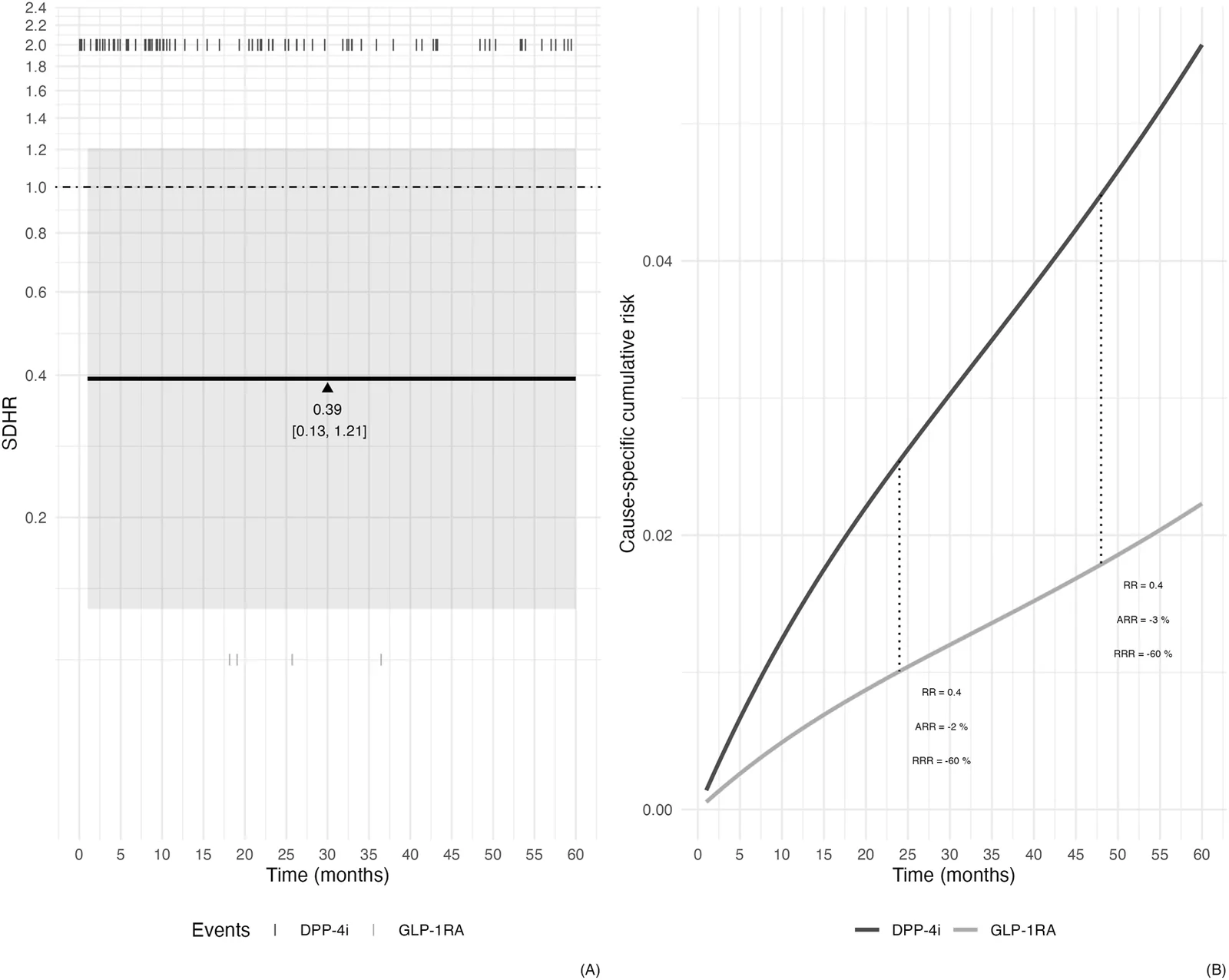
Adjusted competing-risk analysis of cognitive disorders. Cognitive disorder was considered the event of interest, while death without a prior cognitive disorder was treated as a competing event. In **(A)**, the constant sub-distribution hazard ratio estimated with a marginal structural model. In **(B)**, adjusted estimates of crude cumulative estimates of cognitive disorder for DPP-4i and GLP-1RAs groups and the related effect measures computed at 2 and 4 years of the follow-up. Abbreviations: DPP-4i, dipeptidyl peptidase-4 inhibitors; GLP-1RAs, glucagon-like peptide-1 receptor agonists; SDHR: sub-distribution hazard.

In Figure 5B, the estimated risk of cognitive disorders of GLP-1RAs individuals relative to DPP-4i individuals at both 2 and 4 years is 0.4. This corresponds to an absolute risk reduction of 2% and 3%, and a relative risk reduction of 60%.

Turning to the risk of death without a prior diagnosis of cognitive disorders, individuals treated with GLP-1RAs again showed a lower risk compared to those on DPP-4i, as shown in Figure 6. Model-based cumulative incidence estimates show a relative risk of 0.38 for death in the GLP-1RAs group compared to DPP-4i, indicating that GLP-1RAs is associated with less than half the risk. This corresponds to an absolute risk reduction of 7% and a relative risk reduction of 62%. While the hazard-based protective effect diminishes by year five, GLP-1RAs still shows a relative risk of 0.79 at 4 years, with a 6% absolute risk reduction and a 21% relative risk reduction (Figure 7B).

**FIGURE 6:**
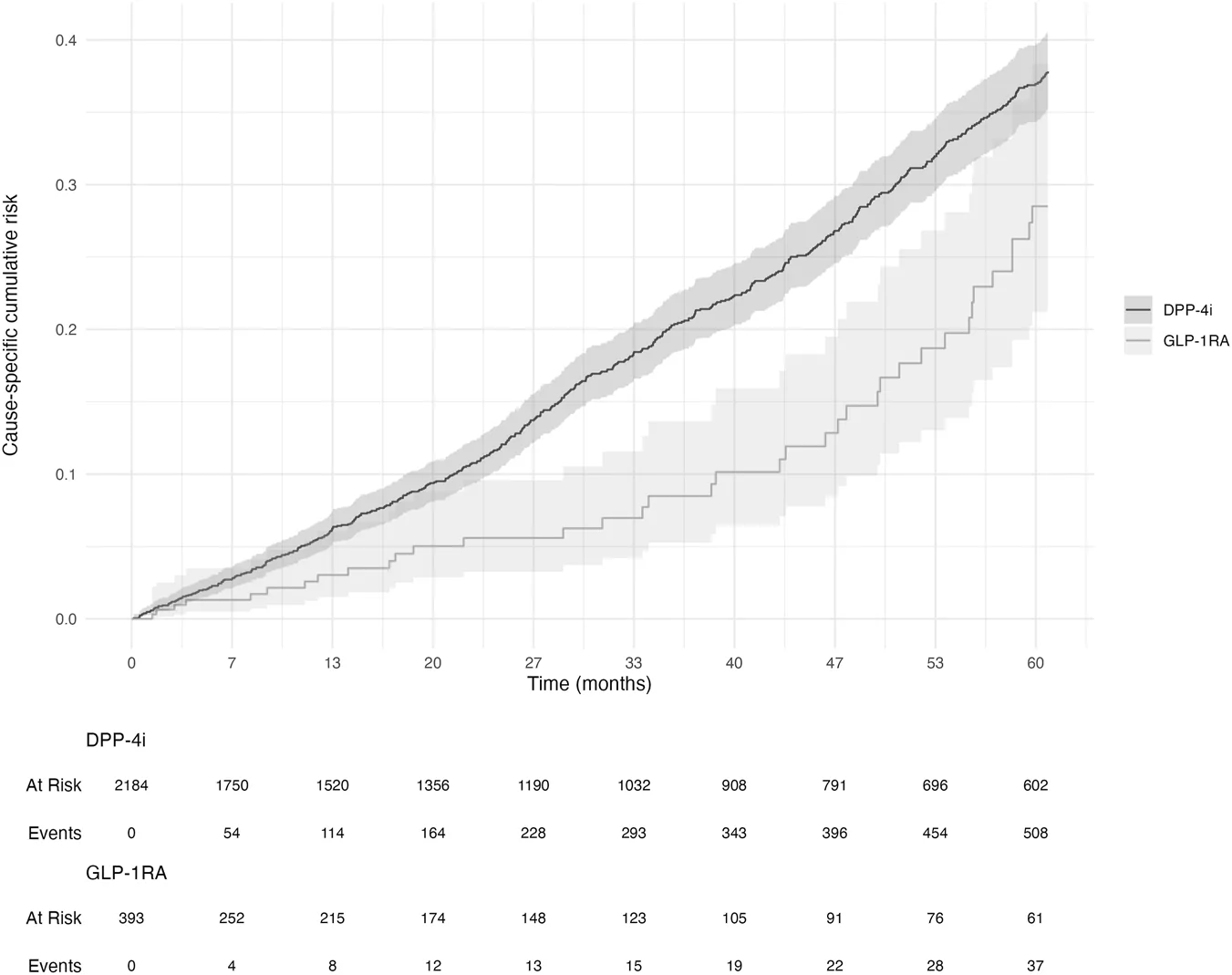
Competing-risk analysis of death without prior cognitive disorder. Death without a prior diagnosis of cognitive disorder was considered the event of interest, while cognitive disorder was treated as a competing event. In unadjusted estimates of crude cumulative risks of death without evidence of cognitive disorder for DPP-4i and GLP-1RAs groups, computed with the Aalen-Johansen estimator. Abbreviations: DPP-4i, dipeptidyl peptidase-4 inhibitors; GLP-1RAs, glucagon-like peptide-1 receptor agonists.

**FIGURE 7:**
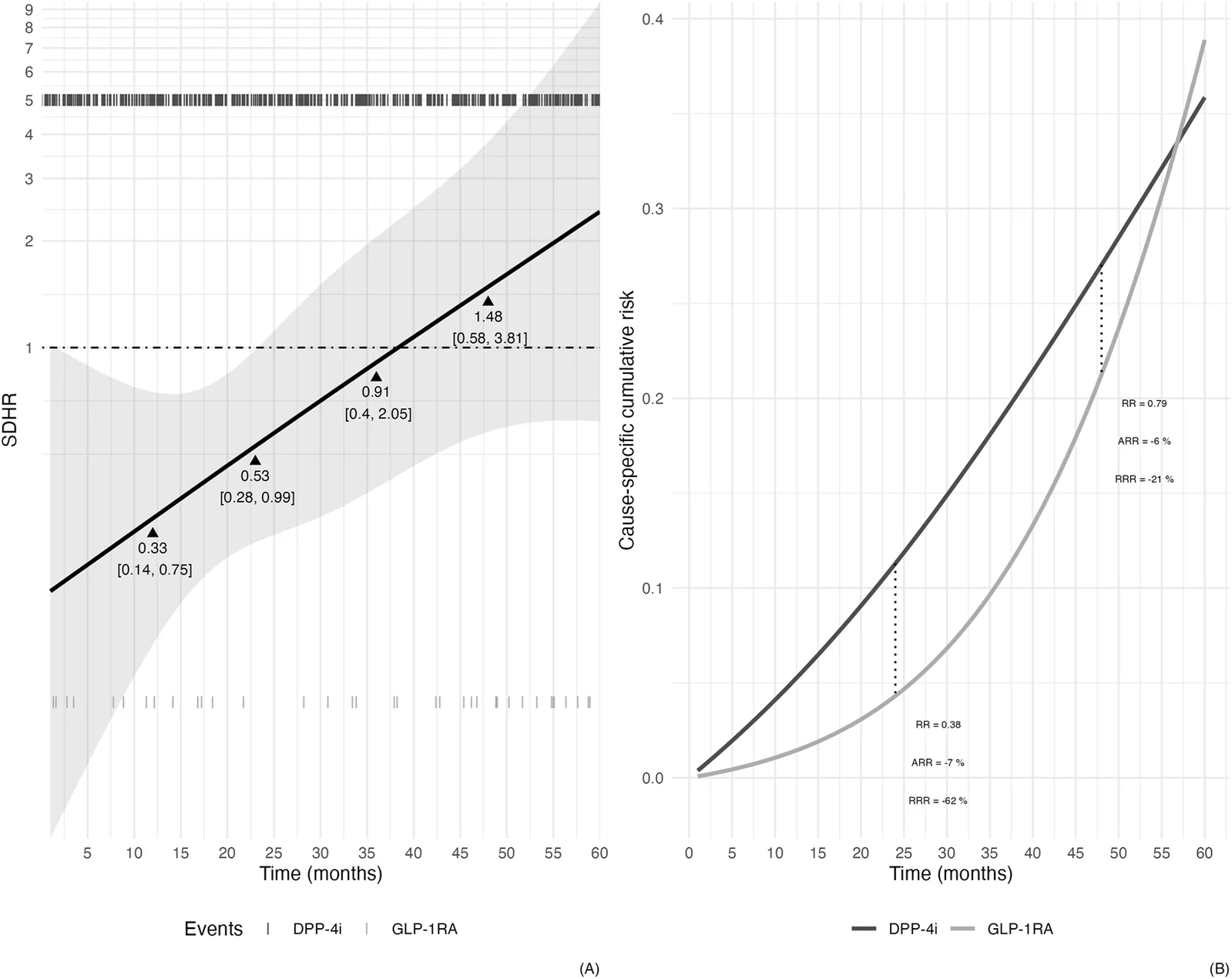
Adjusted competing-risk analysis of death without prior cognitive disorder. Death without a prior diagnosis of cognitive disorder was considered the event of interest, while cognitive disorder was treated as a competing event. In **(A)**, the time-varying sub-distribution hazard ratio estimated with a marginal structural model. In **(B)**, adjusted estimates of crude cumulative risks of death without evidence of cognitive disorder for DPP-4i and GLP-1RAs groups and the related effect measures computed at 2 and 4 years of the follow-up. Abbreviations: DPP-4i, dipeptidyl peptidase-4 inhibitors; GLP-1RAs, glucagon-like peptide-1 receptor agonists; SDHR: sub-distribution hazard.

Figure 7A illustrates the time-varying sub-distribution hazard ratio for GLP-1RAs versus DPP-4i, smoothed using restricted cubic splines with three knots. The time-varying sub-distribution hazard ratio gradually increases from values well below zero - SDHR [95% C.I.], 0.33 [0.14, 0.75], 0.53 [0.28, 0.99], and 0.91 [0.4, 2.05] at 1, 2 and 3 years of follow-up respectively-, eventually crossing the value of 1 toward the end of the 5-year follow-up - SDHR [95% C.I.], 1.48 [0.58, 3.81]-. Assuming adequate control for confounding, this suggests a statistically significant protective effect of GLP-1RAs against death as a first event in the earlier part of the follow-up period.

The intention to treat analysis results (Supplementary Figures S3-S6) are in line with the main results: for the early major cognitive impairment endpoint, GLP-1RA showed a trend of risk reduction (SDHR [95% C.I] 0.57 [0.25, 1.29]), although not statistically significant. The time-varying hazard ratio for the death without prior evidence of major cognitive impairment endpoint was maintained significant, specifically for the first 2 years of follow-up.

Moreover, the sensitivity analyses including calendar year of treatment initiation (Supplementary Figures S9-S12; Supplementary Figures S15-S18) in the weighting model are also in line with the main results. The SDHR for GLP-1RA vs. DPP-4i for the early major cognitive impairment endpoint resulted statistically significant when considering calendar year in the weighting model in the ‘As-treated’ design (SDHR [95% C.I] 0.24 [0.08, 0.68]) but not when including it in the ITT design (SDHR [95% C.I] 0.47 [0.21, 1.04]).

## 4 Discussion

In this retrospective cohort study of elderly Italian patients with T2DM, initiation of GLP-1RAs, compared to DPP-4i, was linked to a lower risk of a composite outcome of cognitive disorders or death. Notably, this is the first study to account for the competing risk of death when examining cognitive decline in older adults treated with these therapies, despite a growing body of literature (De Giorgi et al., 2025a). Overall, patients on GLP-1RAs had a 66% relative risk reduction vs. DPP-4i for the composite outcome in the first few years, though this benefit declined over time and was no longer significant by year 5.

Although the absolute incidence of cognitive disorders was low in both groups, reflecting the relatively short window of follow-up for neurodegenerative outcomes, the GLP-1RAs group consistently showed a lower cumulative incidence. Notably, only four individuals in the GLP-1RAs cohort developed cognitive disorders, compared to 85 in the DPP-4i group. The estimated trend showed an SDHR of 0.4 translated into relative risk reduction of cognitive disorders of approximately 60% in both two and 4 years. These findings should be interpreted with caution because of the very small number of cognitive disorder events among GLP-1RA initiators and the resulting imprecision of the estimates. In particular, the confidence interval for the sub-distribution hazard ratio included the null value. Therefore, these results do not provide sufficient evidence to conclude that GLP-1RAs have a protective or neuroprotective effect on cognitive outcomes. Rather, the direction of the association may be considered hypothesis-generating and warrants confirmation in larger studies with a greater number of cognitive events and, ideally, cognitive-specific assessments. However, our results are consistent with the growing body of evidence suggesting that GLP-1RAs may have neuroprotective effects, possibly via anti-inflammatory and neurotrophic pathways. For instance, a recent target trial emulation using U.S. administrative data showed a significant 33% reduced risk of Alzheimer’s disease and related dementias (ADRD) in GLP-1RAs users versus other glucose-lowering drugs, including DPP-4i, over 8 years (Adjusted HR [95% CI], 0.67 [0.47–0.96]), with no significant difference for mild cognitive impairment (Tang et al., 2025).

Similarly, a large Swedish nationwide cohort revealed that GLP-1RAs initiation was associated with a significantly lower dementia risk compared to sulfonylureas and DPP-4i, with consistent findings across metformin-only and insulin user subgroups. Both intention-to-treat and per-protocol analyses, showed hazard ratios between 0.38 and 0.78, supporting the protective effect of GLP-1RAs regardless of baseline treatment (Tang et al., 2024).

A Danish register-based study also reported a higher risk of hospitalization for major cognitive impairment in DPP-4i users versus GLP-1RAs (HR: 1.58; 95% CI: 1.22–2.06) (Battini et al., 2024), aligning with two U.S. observational studies showing a 28% lower hazard of cognitive deficits within the first year of semaglutide use compared to sitagliptin (HR: 0.72; 95% CI: 0.64–0.80) (De Giorgi et al., 2025b) and a lower risk of several neurocognitive disorders when using GLP-1RAs compared to DPP-4i (HR: 0.90; 95% CI: 0.87–0.94), reinforcing the broader neuropsychiatric benefit profile of this drug class (Xie et al., 2025).

In our study, GLP-1RAs were also associated with a reduced risk of death in patients without prior major cognitive impairment. As with the composite endpoint, this survival advantage appeared time-dependent, with the strongest effect observed within the first 2 years (relative risk reduction of 62%). While the magnitude of the effect decreased over time, a residual benefit persisted over 4 years. These findings support the idea that GLP-1RAs may provide systemic benefits beyond glucose control, potentially improving medium-term survival in older adults with diabetes.

Most cognitive disorder events in the DPP-4i group appeared vascular in origin, whereas they were less frequent among GLP-1RAs users, consistent with the vascular protective effects and broader cardiometabolic benefits of GLP-1RAs (Lenharo, 2023; Husain et al., 2019; Giugliano et al., 2022; Gilbert and Pratley, 2020). Given that cerebrovascular disease is a key contributor to vascular dementia, this observation provides a biologically plausible explanation for the differential risk of cognitive decline across drug classes. However, administrative claims data, lacking neuroimaging confirmation, have a low positive predictive value for accurately subtyping dementia, thereby limiting our ability to confidently differentiate vascular dementia from Alzheimer’s disease or mixed pathologies. Moreover, since hospital discharge records mainly capture severe vascular-related events, milder impairments such as early memory or executive deficits were likely underestimated. A Danish study similarly reported increased risk only for major cognitive outcomes (Battini et al., 2024). Future research with validated cognitive scales and neuropsychological tests is needed to fully characterize these associations.

Overall, our findings suggest that GLP-1RAs may reduce cognitive decline and mortality in elderly patients with T2DM, particularly in the early treatment years. The composite endpoint captures competing risks in this vulnerable population, while separate analyses clarify their impact on aging and cognitive health.

The use of administrative data did not allow adjustment for important clinical variables such as diabetes duration, BMI, blood pressure, and frailty. Although inverse probability weighting and high-dimensional propensity score methods achieved covariate balance for all measured variables, residual confounding cannot be excluded, as factors such as education, socioeconomic status, baseline cognition, and lifestyle were not captured in this administrative database, and baseline comparability could only be assessed based on available data. Further studies with longer follow-up and detailed cognitive assessments are warranted to confirm and generalize these findings. While our analysis is restricted to a single Italian province, it may reflect broader national prescribing patterns: for example, a large Lombardy cohort (>2 million over 65s) also reported low GLP-1RAs initiation between 2012 and 2018 ^14^, consistent with our results and suggesting applicability to other regions. Although we tried to control for temporal changes in patient characteristics, treatment patterns, and diabetes management including calendar year of treatment initiation in the weighting model in the sensitivity analyses, residual confounding cannot be excluded.

The attenuation of the treatment effect over time should be interpreted cautiously, as several epidemiological mechanisms may contribute to this pattern. Although residual healthy initiator bias cannot be fully ruled out, the E-value of 6.35 (lower confidence limit: 2.72) suggests that relatively strong unmeasured confounding would be required to fully explain the observed early association. Furthermore, the progressive attenuation of the hazard ratio is highly characteristic of a ‘depletion of susceptibles’ (frailty effect), wherein individuals most vulnerable to the outcome experience the event early, leaving a progressively lower-risk pool of survivors. An initial healthy initiator effect could also artificially inflate early apparent benefits; however, our sensitivity analyses adjusting for calendar year of treatment initiation, which should partially control for evolving clinical practices and temporal channeling biases, did not explained this ‘early’ protection. Nevertheless, since the observational nature of the data, although a true time-dependent biological effect of GLP-1RAs cannot be excluded, the present study cannot distinguish between these alternative explanations. Therefore, the observed attenuation of the treatment effect should not be interpreted as evidence of waning biological efficacy.

## 5 Conclusion

In this real-world cohort of elderly patients with T2DM, GLP-1RAs initiation was associated with a lower early risk of the composite outcome of mortality or cognitive disorders compared with DPP-4i, with the observed benefit appearing to be largely driven by lower mortality. Cognitive-specific findings were limited by the very small number of events and should therefore be considered exploratory and hypothesis-generating, rather than evidence of a neuroprotective effect. These results highlight the importance of considering competing mortality risks when assessing long-term outcomes in frail populations.

## Data Availability

The datasets presented in this article are not readily available because the scientific work for this project was coordinated by Università degli Studi di Milano in collaboration with the Pharmaceutical Department of ASL BT, Trani, Italy. The data that support the findings of this study are available from the Italian province participating to the study, but restrictions apply to the availability of these data, which were used under license (as by third-party sources) for the current study and are not publicly available. However, data are available with permission of Italian province, which is the data owner. The local data used for this study are not publicly available. Requests to access the datasets should be directed to area.farmaceutica@aslbat.it.
