# Supplementary material for: Exploring the impact of GLP-1 analogues and DPP-4 inhibitors on cognitive decline in elderly patients with type 2 diabetes: a competing risks analysis from a real-world cohort
Source: Front Pharmacol. 2026 Oct 15;17:1822158. doi: 10.3389/fphar.2026.1822158 (PMC13716640; doi:10.3389/fphar.2026.1822158)
Supplement: Supplementary file 1 [file Supplementaryfile1.docx]

# Sensitivity Analysis 1: Intention To Treat analysis

### 1. Composite Endpoint (Early Major Cognitive Impairment and All-Cause Mortality)

The cumulative incidence analysis (**Supplementary Figure 1**) demonstrates a clear and early divergence between the two treatment cohorts. Patients initiating DPP-4 inhibitors experience a steadily increasing cumulative risk of the cognitive endpoint, reaching approximately 30% at the 60-month mark. In contrast, the GLP-1RA cohort maintains a substantially lower trajectory throughout the observation period, culminating in a cumulative risk of roughly 12%. The at-risk table confirms adequate follow-up density, with 610 events captured in the DPP-4i arm and 49 events in the GLP-1RA arm by the end of the 5-year period.

**Supplementary Figure 1**

Panel A of **Supplementary Figure 2** highlights that as time progresses, the Absolute Risk Reduction (ARR) continues to grow, widening from -7% at roughly 24 months to -10% at 48 months, reflecting the ongoing accumulation of prevented events in the GLP-1RA group. However, the Relative Risk Reduction (RRR) shows the opposite trend, attenuating from -63% to -47% over the same timeframe.

Panel B of **Supplementary Figure** shows that the treatment effect violates the proportional hazards assumption and is explicitly time varying. The protective association is exceptionally strong in the first year, with a Hazard Ratio (HR) of 0.35 (95% CI: 0.19–0.63) at 12 months. This effect gradually drifts towards the null over time, reaching an HR of 0.84 (95% CI: 0.38–1.84) at 48 months, where the upper bound of the confidence interval crosses the threshold of non-significance after approximately 30 months.

**Supplementary Figure 2**

### 2. Cause-Specific Endpoint: Early Major Cognitive Impairment

**Supplementary Figure 3**

The analysis of early major cognitive impairment, presented in **Supplementary Figure 3**, reveals a more subtle divergence between the treatment arms compared to the broader dementia endpoint. The cause-specific cumulative risk for the DPP-4i cohort rises gradually, reaching approximately 4.5% by the end of the 60-month follow-up. In contrast, the GLP-1RA cohort exhibits a lower incidence trajectory, arriving at a cumulative risk of roughly 2.5% at 60 months.

It is crucial to note the significantly lower event rate for this specific endpoint. As detailed in the at-risk table, only 96 events were recorded in the DPP-4i arm and a mere 9 events in the GLP-1RA arm over the 5-year period. This scarcity of events is visually reflected in the wider confidence intervals and the more pronounced "step-like" progression of the GLP-1RA incidence curve.

**Supplementary Figure 4**

Panel A of **Supplementary Figure 4** illustrates that the RR remains entirely stable throughout the observation period, calculated at 0.57 at both the 24-month and 48-month marks. Consequently, the RRR remains constant at -43%. Because of the overall low incidence of this early endpoint, the ARR is minimal, stabilizing at a constant -1% across the evaluated time points.

In Panel B of **Supplementary Figure 4** unlike the time-varying hazard observed for the broader dementia outcome, the SDHR for early major cognitive impairment is modeled as constant over time. The estimated SDHR is 0.57 (95% CI: 0.25–1.29).

While the point estimate suggests a substantial 43% reduction in the sub-distribution hazard of early major cognitive impairment for GLP-1RA users relative to DPP-4i users, the wide confidence interval which clearly crosses the null value of 1.0 indicates that this association does not reach statistical significance.

### 3. Cause-Specific Endpoint: Death with No Prior Evidence of Major Cognitive Impairment

**Supplementary Figure 5**

The analysis of death with no prior evidence of major cognitive impairment, depicted in **Supplementary Figure 5**, reveals a pronounced and early divergence between the two treatment arms. In this competing risk framework, the cause-specific cumulative risk of mortality for the DPP-4i cohort climbs steeply, reaching approximately 25% by the end of the 60-month follow-up. Conversely, the GLP-1RA cohort demonstrates a markedly suppressed mortality trajectory, reaching a cumulative risk of only 10% at 60 months. The at-risk table highlights a robust event count for this endpoint, with 514 deaths recorded in the DPP-4i arm and 40 deaths in the GLP-1RA arm.

**Supplementary Figure 6**

Panel A of **Supplementary Figure 6** illustrates the ARR) steadily widening from -6% at 24 months to -9% at 48 months, reflecting the continuous accumulation of prevented (or delayed) deaths in the GLP-1RA group. Conversely, the RRR follows an attenuating trajectory, decreasing from an initial -70% at 24 months to -49% by 48 months.

Panel B of **Supplementary Figure 6** shows that the protective association against mortality is exceptionally strong during the first two years, with an SDHR of 0.27 (95% CI: 0.12–0.60) at 12 months and 0.41 (95% CI: 0.22–0.76) at 24 months. This advantage progressively drifts toward the null, crossing the threshold of non-significance shortly after 24 months, and ultimately reaching an SDHR of 0.91 (95% CI: 0.38–2.19) at 48 months.
