# Supplementary material for: Exploring the impact of GLP-1 analogues and DPP-4 inhibitors on cognitive decline in elderly patients with type 2 diabetes: a competing risks analysis from a real-world cohort
Source: Front Pharmacol. 2026 Oct 15;17:1822158. doi: 10.3389/fphar.2026.1822158 (PMC13716640; doi:10.3389/fphar.2026.1822158)
Supplement: Supplementary file 2 [file Supplementaryfile2.docx]

# Sensitivity Analysis 2: addition of calendar year in the propensity score model to balance for shifting clinical practices during the study period

### 1. Composite Endpoint (Early Major Cognitive Impairment and All-Cause Mortality)

In the calendar-year adjusted model, the cumulative incidence of the composite endpoint at 60 months approaches 45% for the DPP-4i arm and approximately 30% for the GLP-1RA arm. The at-risk table demonstrates a robust overall sample, with 593 events captured in the DPP-4i cohort and 41 events in the GLP-1RA cohort by the end of the follow-up period.

**Supplementary Figure 7**

The Absolute Risk Reduction (ARR) widens progressively from -9% at 24 months to -16% at 48 months, illustrating the continuous accumulation of prevented events. In contrast, the Relative Risk Reduction (RRR) attenuates over time, decreasing from -69% to -51% across the same interval. The time-varying Hazard Ratio (HR) confirms a profound early protective association (HR 0.35, 95% CI: 0.18–0.67 at 24 months) that gradually drifts toward the null, crossing the threshold of non-significance around month 37 and reaching an HR of 0.78 (95% CI: 0.34–1.79) at 48 months. This non-proportionality perfectly captures the "depletion of susceptibles" phenomenon, even after rigorously adjusting for temporal shifts in prescribing guidelines.

**Supplementary Figure 8**

### 2. Cause-Specific Endpoint: Early Major Cognitive Impairment

The cause-specific cumulative risk for early major cognitive impairment reaches approximately 6% for the DPP-4i cohort and 2.5% for the GLP-1RA cohort at the 60-month mark. Notably, this specific endpoint is characterized by extreme sparsity, with 85 events recorded in the DPP-4i arm and only 4 events in the GLP-1RA arm.

**Supplementary Figure 9**

Unlike the composite endpoint, the Subdistribution Hazard Ratio (SDHR) for early major cognitive impairment is constant over time, estimated at 0.24. The inclusion of the calendar year in the propensity score model has narrowed the 95% confidence interval (0.08–0.68), rendering this profound 76% relative risk reduction statistically significant. Correspondingly, the RRR remains constant at -76%, while the ARR stays minimal (-2% at 24 months, -3% at 48 months) due to the overall rarity of the outcome. Given the reliance on only 4 events in the active treatment arm, this statistically significant result must be interpreted with strict methodological caution regarding small-sample variance approximations.

**Supplementary Figure 10**

### 3. Cause-Specific Endpoint: Death with No Prior Evidence of Major Cognitive Impairment

For the competing risk of mortality without prior dementia, the cumulative risk climbs to approximately 38% for the DPP-4i cohort compared to roughly 28% for the GLP-1RA cohort at 60 months. The statistical power for this competing event is high, driven by 508 deaths in the DPP-4i group and 37 deaths in the GLP-1RA group.

**Supplementary Figure 11**

The ARR for mortality steadily increases from -7% at 24 months to -13% at 48 months, while the RRR attenuates from -64% to -46% over the same timeframe. The SDHR analysis reveals a strongly time-varying protective effect against mortality, with a pronounced early survival benefit (SDHR 0.38, 95% CI: 0.20–0.74 at 24 months). Similar to the composite outcome, this survival advantage progressively weakens, crossing into non-significance and reaching an SDHR of 0.90 (95% CI: 0.37–2.22) at 48 months. This confirms that the time-varying nature of the composite endpoint is predominantly driven by the mortality component and the inherent frailty dynamics of this older diabetic cohort.

**Supplementary Figure 12**
