# Supplementary material for: Exploring the impact of GLP-1 analogues and DPP-4 inhibitors on cognitive decline in elderly patients with type 2 diabetes: a competing risks analysis from a real-world cohort
Source: Front Pharmacol. 2026 Oct 15;17:1822158. doi: 10.3389/fphar.2026.1822158 (PMC13716640; doi:10.3389/fphar.2026.1822158)
Supplement: Supplementary file 3 [file Supplementaryfile3.docx]

# Sensitivity Analysis 3: addition of calendar year in the propensity score model to balance for shifting clinical practices during the study period – ITT analysis

### 1. Composite Endpoint (Early Major Cognitive Impairment and All-Cause Mortality)

Under the ITT framework, the cumulative incidence trajectories mirror the primary analysis. The DPP-4i cohort reaches a cumulative risk of nearly 30% at 60 months, compared to approximately 12% in the GLP-1RA cohort. The ARR widens from -7% to -12% between 24 and 48 months. The HR demonstrates the hallmark attenuation associated with frailty dynamics in older populations. The early protective effect (HR 0.39, 95% CI: 0.21–0.71 at 24 months) gradually drifts toward the null, crossing into non-significance around month 35 (HR 0.71, 95% CI: 0.35–1.47 at 48 months)

**Supplementary Figure 13**

**Supplementary Figure 14**

### 2. Cause-Specific Endpoint: Early Major Cognitive Impairment

The ITT framework successfully captures events that occurred after treatment discontinuation. Consequently, the event count in the GLP-1RA arm increases from 4 (in the As-Treated model) to 9 events. With this slight increase in events, the Subdistribution Hazard Ratio (SDHR) stabilizes at 0.47. Crucially, the 95% confidence interval (0.21–1.04) now crosses 1.0, rendering the association statistically non-significant. This definitively confirms that the profound, statistically significant effect (SDHR 0.24) observed in the As-Treated calendar-year adjusted model was an artifact driven by the breakdown of small-sample variance approximations when relying on only 4 events.

**Supplementary Figure 15**

**Supplementary Figure 16**

### 3. Cause-Specific Endpoint: Death with No Prior Evidence of Major Cognitive Impairment

The mortality trajectories remain consistent, with the DPP-4i arm reaching a cumulative risk of roughly 25% versus 10% in the GLP-1RA arm at 60 months. The SDHR perfectly tracks the time-varying nature of the composite endpoint. The initial survival benefit (SDHR 0.36 at 24 months) attenuates and becomes non-significant by month 36 (SDHR 0.49, 95% CI: 0.22–1.09). This confirms that the attenuation in the composite endpoint is primarily driven by the mortality component and the subsequent equalization of surviving risk pools.

**Supplementary Figure 17**

**Supplementary Figure 18**
