# Supplementary material for: Exploring the impact of GLP-1 analogues and DPP-4 inhibitors on cognitive decline in elderly patients with type 2 diabetes: a competing risks analysis from a real-world cohort
Source: Front Pharmacol. 2026 Oct 15;17:1822158. doi: 10.3389/fphar.2026.1822158 (PMC13716640; doi:10.3389/fphar.2026.1822158)
Supplement: Supplementary file 4 [file Table1.docx]

**Supplementary Table S1:** ICD codes for cognitive disorders

| **ICD-9-CM** | **ICD-10-CM** | **Description** |
| --- | --- | --- |
| 331.0 | G30.9 | Alzheimer’s dementia |
| 290.0 | F03.90 | Senile dementia, uncomplicated |
| 290.10 | F03.90 | Presenile dementia, uncomplicated |
| 290.11 | F03.90 | Presenile dementia with delirium |
| 290.12 | F03.90/F05 | Presenile dementia with delusional features |
| 290.13 | F03.90 | Presenile dementia with depressive features |
| 290.2 | - | Senile dementia with delusional features |
| 290.20 | F03.90/F05 | Senile dementia with delusional features |
| 290.21 | F03.90 | Senile dementia with depressive feature |
| 290.3 | F03.90/F05 | Senile dementia with delirium |
| 290.4 | - | Vascular dementia, uncomplicated |
| 294.40 | F01.50 | Vascular dementia, uncomplicated |
| 290.41 | F01.51 | Vascular dementia, with delirium |
| 290.42 | F01.51 | Vascular dementia, with delusions |
| 290.43 | F01.51 | Vascular dementia, with depressed mood |
| 290.8 | F03.90 | Unspecified dementia, without behavioural disturbance |
| 290.9 | F03.90 | Unspecified dementia, without behavioural disturbance |
| 294.1 | - | Dementia in conditions classified elsewhere w/out behavioural disturbance |
| 294.10 | F02.80 | Dementia in conditions classified elsewhere |
| 294.11 | F02.81 | Dementia in conditions classified elsewhere |
| 331.2 | G31.1 | Senile degeneration of the Brain |
| 331.11 | G31.01 | Pick’s disease |
| 331.19 | G31.09 | Other frontotemporal dementia |
| 331.82 | G31.83 | Dementia with Lewy bodies |
| 331.83 | G31.84 | Mild cognitive impairment, so stated |
